# Categorical Versus Graded Beliefs

**DOI:** 10.3389/fpsyg.2022.817940

**Published:** 2022-03-21

**Authors:** Franz Dietrich

**Affiliations:** Paris School of Economics, CNRS, Paris, France

**Keywords:** logic vs. rational choice theory, yes/no belief vs. graded belief, subjective probability, belief binarization, lottery paradox, impossibility theorem, binary belief, credence

## Abstract

This essay discusses the difficulty to reconcile two paradigms about beliefs: the binary or categorical paradigm of yes/no beliefs and the probabilistic paradigm of degrees of belief. The possibility for someone to hold beliefs of both types simultaneously is challenged by the lottery paradox, and more recently by a general impossibility theorem by Dietrich and List. The nature, relevance, and implications of the tension are explained and assessed.

## 1. Two Types of Belief and Their Potential Coexistence

Rational choice theory and logic have very different concepts of belief, each of which enjoys significant appeal and wide applications. Rational choice theory takes agents to have graded beliefs of the form of subjective probability assignments. One might believe that it rains with subjective probability 2/3, or that one will stay healthy with subjective probability 3/4. By contrast, logic takes agents to have categorical beliefs, of the form of “yes” or “no” (or abstention). One might believe that it rains, or that one will stay healthy, in a categorical rather than graded sense. Believing something categorically should not be confused with complete certainty, i.e., with maximal graded belief: otherwise one would hardly ever believe anything in the categorical sense.

The advantage for rational choice theory of assuming probabilistic beliefs is considerable: it opens to the door to the classic notion of a rational agent seeking to maximise expected utilities, since expected utilities are the result of combining the probabilistic model of beliefs with the utility-based model of goals, values, or desires. As such, probabilistic beliefs form an intrinsic part of the classic homo oeconomicus. By contrast, logicians are less interested in decision making, and, hence, do not need to combine beliefs with goals, values, or desires. Instead, they often focus on beliefs alone, which they usually take to be truth-oriented, logically consistent, and deductively closed, and to evolve *via* reasoning and belief revision. Categorical beliefs lend themselves to reasoning and belief revision, as logicians have amply demonstrated.

Of course, rational choice theory has its own theory of belief revision: a highly unified Bayesian theory, in which probabilistic beliefs undergo Bayesian updating as new information arrives. But it is questionable whether Bayesianism yields a theory of reasoning as opposed to revision, and more generally whether probabilistic beliefs and reasoning go well with one another. Reasoning differs fundamentally from revision, by drawing not on new information but on inferences from existing beliefs. For logicians, reasoning happens in language, and is a process of drawing conclusions from initially believed premises. Reasoning works much more naturally with categorical than with graded beliefs.

Rational choice theorists and logicians are both right in their own terms, since both models of belief fulfill the purpose set by the respective discipline. But can both kinds of belief coexist in the same agent? Such an agent would for instance simultaneously believe that it rains with subjective probability 2/3 and that it rains *simpliciter*. More generally, for any relevant proposition *p*, the agent would hold some subjective probability of *p* and some yes/no belief about *p*. Depending on the context, the agent might draw either on their categorical beliefs or on their graded beliefs. In some contexts, the agent might reason logically with categorical beliefs, by drawing inferences from existing beliefs, thereby forming new beliefs. When learning information, the agent might on the one hand logically revise categorical beliefs, and on the other hand Bayes-revise graded beliefs. In decision-making contexts, the agent might either use a simple heuristic based on categorical beliefs and values, or use a more sophisticated decision rule (possibly the expected-utility rule) based on graded beliefs and values. In short, each type of belief would play a different functional role. Neither type would be redundant, since each type is tailored to its own role, and each type outperforms the other in its own area of application. Under this attractive division-of-labor picture, both belief types would be legitimate components of psychology.

But this ecumenical picture can only be maintained if the two belief types are mutually compatible in some sense, i.e., can coexist coherently. What exactly coherence amounts to is a question on its own. *Prima facie*, one would expect the agent to categorically believe propositions in which they have high degree of belief, and to categorically disbelieve propositions in which they have low degree of belief. This has come to be known as the “Lockean Thesis” (Foley, 2009). As we shall however see, this thesis leads straight into the “lottery paradox,” from where an active literature unfolds about whether and how both belief types are co-tenable.

## 2. From the Lottery Paradox to a General Impossibility Theorem About Coexistence of Both Belief Types

Our notion of “can coexist” is normative, not positive. That is, we do not describe real agents, but ask whether an idealised agent—perhaps called a “rational” agent—can hold both belief types. The coexistence of both belief types is challenged by the well-known *lottery paradox* (Kyburg, 1961). This paradox starts from the Lockean Thesis—whereby one believes a proposition categorically if and only if one has a high enough degree of belief in it—and shows that this thesis generates a serious problem: even when graded beliefs are perfectly rational, i.e., obey probability theory, the corresponding set of categorical beliefs, formed *via* the Lockean Thesis, can be irrational, i.e., neither consistent nor deductively closed.

Why? In the lottery paradox, you are given a book of 100 pages. You know that exactly one page is black and all others are white. You have no idea about which page is black. So for each page you have a subjective probability of 99/100 that it is white. This subjective probability is high enough to make you (categorically) believe that the page is white. Meanwhile you have a subjective probability of 1 that not all pages are white. This maximal subjective probability is of course high enough to make you (categorically) believe that not all pages are white. Your categorical beliefs present two logical flaws. For one, you believe that the first page is white, that the second page is white, and so on, but you fail to believe an implication of these 100 beliefs, namely that all pages are white—a violation of deductive closure. Worse, you believe the opposite of this implication, namely that *not* all pages are white—a violation of logical consistency.

Though special in its setup, the lottery paradox highlights a deep and general problem. The literature has responded to it in different ways. One approach is “constructive” and consists in introducing, defending, or criticising concrete non-Lockean relations between both belief types that avoid the paradox. A number of potential relations are on the table; see for instance the “odds-threshold rule” in Lin and Kelly (2012a,b), the “stability theory” in Leitgeb (2014, 2017), and the “premise-based,” “distance-based,” “sequential,” “relevance-based,” and “holistic-threshold-based” relations in Dietrich and List (2018, 2021). Douven and Rott (2018) critically analyse the first two mentioned proposals. Earlier work about the lottery paradox includes (Hawthorne and Bovens, 1999; Douven and Williamson, 2006; Douven and Romeijn, 2007).

Taking an axiomatic rather than constructive approach, the lottery paradox was recently generalised into an impossibility theorem, proved in two versions by Dietrich and List (2018, 2021). Other impossibility theorems generalising the paradox were proved by Schurz (2019).

We here sketch Dietrich and List's theorem. It says: There is *no* form of coexistence of both belief types that respects certain initially plausible conditions. What are these conditions? There are six of them. The first three pertain each to one belief type only, and the next three pertain to the relationship between both belief types. Here are informal statements of the conditions:

1. The agent only ever holds categorical beliefs that are consistent and deductively closed.
2. The agent only ever holds degrees of belief that are probabilistically coherent (so that, for instance, the probability of “rain or snow” is the sum of the probabilities of “rain” and “snow.”)
3. Any (probabilistically coherent) degrees of belief are allowed, i.e., can be held jointly with at least some categorical beliefs.
4. Whenever a proposition is believed with subjective probability 1, then it is believed categorically.
5. The two belief types impose at least some non-trivial constraints on one another, rather than being essentially independent of one another.
6. Any dependence between the two belief types is “local” (“proposition-wise”) rather than ‘global’ (“holistic,”) in a sense defined below. For instance, the Lockean Thesis postulates a purely local dependence, since the categorical belief in a proposition depends solely on the degree of belief in *this* proposition.

To state these conditions more precisely, let me sketch the formal setup. Consider a set *X* of propositions (or events) of interest; in the lottery paradox, *X* contains at least propositions about page colors. *X* could be very large, possibly containing *all* meaningful propositions, or very small, possibly containing only propositions about a particular topic such as page colors, the Corona virus, or tomorrow's weather^1^. The agent's graded beliefs are represented by a *degree-of-belief function* *Pr* that assigns to each proposition *p*∈*X* a subjective probability *Pr*(*p*)∈[0, 1]. The agent's categorical beliefs are represented by a *belief set* *B*⊆*X*, containing the (categorically) believed propositions. Certain combinations (*Pr, B*) of a degree-of-belief function and a belief set are “coherent” or “(rationally) co-tenable,” the others are not. Formally, coherence or co-tenability defines a binary relation between degree-of-belief functions *Pr* and belief sets *B*—the relation of being mutually coherent or co-tenable.

The theorem assumes that this coherence relation satisfies six conditions. They were stated informally above. Here, are more formal re-statements:

1. Categorical beliefs are logically coherent: all permissible belief sets *B* are logically consistent and deductively closed. “Permissible” means that *B* is coherent with at least one degree-of-belief function *Pr*. Deductive closedness is defined relative to *X*: every proposition *from* *X* that *B* entails is contained in *B*.
2. Graded beliefs are probabilistically coherent: any permissible degree-of-belief function *Pr* obeys the laws of probability. “Permissible” means that *Pr* is coherent with at least one belief set *B*.
3. No coherent graded beliefs are ruled out: every probabilistically coherent degree-of-belief function *Pr* is permissible. “Permissible” was just defined.
4. Completely certain propositions are categorically believed: for any coherent combination (*Pr, B*) and any proposition *p*∈*X*, if *Pr*(*p*) = 1 then *p*∈*B*.
5. The two belief types are non-loosely related: at least one (permissible) degree-of-belief function *Pr* requires to believe some proposition *p*∈*X* that is not completely certain, i.e., satisfies *Pr*(*p*)≠1. This rules out that all categorical beliefs are optional except under complete certainty. Technically, a degree-of-belief function *Pr* is said to “require” to believe a proposition *p* if *p* is contained in all belief sets coherent with *Pr*.
6. Any dependence between both belief types is “local” or “proposition-by-proposition:” whether the graded beliefs *require* to believe a given proposition only depends on the graded belief in *this* proposition (where “require to believe” was just defined). For instance, if the graded beliefs require to believe in rain, then changing the degree of belief in sunshine without changing the degree of belief in rain does not lift the requirement to believe in rain. The Lockean Thesis is an example of locality: here, believing a proposition is required if and only if the degree of belief in this proposition is high enough.

The impossibility theorem says: these six conditions are mutually incompatible^2^.

A special kind of coherence relation deserves being mentioned: so-called *functional* or *deterministic* relations, in which the graded beliefs fully determine the categorical beliefs. Formally, functionally means that each permissible degree-of-belief function *Pr* is coherent with exactly one belief set *B*. Such a functional relation can be captured by a *binarization function* *f* which maps any (permissible) degree-of-belief function *Pr* to the corresponding belief set *B* = *f*(*Pr*). The mentioned impossibility result was initially stated under the assumption of functionality, hence as a theorem about the inexistence of any binarization *function* satisfying certain conditions (Dietrich and List, 2018). To our later surprise, the impossibility extends to the much broader case without functionality assumption (Dietrich and List, 2021). The non-functional case allows one's categorical beliefs to be related much more loosely to one's graded beliefs: one's degrees of belief could impose almost no constraints on categorical beliefs, thereby leaving much freedom in what to believe categorically. Despite such freedom, it remains impossible to hold both belief types in accordance with the mentioned conditions.

## 3. What to Make of This Impossibility?

Different reactions to the impossibility theorem are imaginable. Either one takes rational agents to have only graded beliefs, no categorical beliefs—against the logical paradigm. Or one takes rational agents to have only categorical beliefs, no graded beliefs—against the rational-choice-theoretic paradigm. Or one maintains coexistence, but gives up some of the conditions assumed in the incompatibility theorem. As a matter of fact, most conditions seem inescapable. But there are two important exceptions:

- One might give up the locality of the dependence between both belief types (condition 6). This would in particular give up the Lockean Thesis. Although locality is less demanding than the Lockean Thesis—it for instance does not imply functionality—locality is a strong constraint, normatively and mathematically, so that sacrificing it might be in order. Examples of non-local (“holistic”) relations between both belief types are the mentioned relations in Lin and Kelly (2012a,b), Leitgeb (2014, 2017), or Dietrich and List (2018, 2021). Some of these relations are functional, others are non-functional.
- More radically, one could turn to a different theory of graded beliefs, by giving up probabilistic beliefs in favor of some other notion of graded belief. Multi-valued logic and ranking theory provide alternative kinds of graded belief. This intervention goes beyond giving up condition 2, since it alters the formal object of a degree of belief, and hence the range of degree-of-belief functions (initially, the set [0, 1]). Interestingly, ranking-theoretic beliefs (Spohn, 2012) would escape the impossibility and allow for a viable coexistence of graded and categorical beliefs—even a functional one. Needless to say, orthodox rational choice theory would be reluctant to replace “their” probabilistic paradigm by an altogether different, albeit graded, notion of belief.

## Author Contributions

The author confirms being the sole contributor of this work and has approved it for publication.

## Funding

This work was supported by the French Research Agency through the Grants ANR-17-CE26-0003, ANR-16-FRAL-0010, and ANR-17-EURE-0001.

## Conflict of Interest

The author declares that the research was conducted in the absence of any commercial or financial relationships that could be construed as a potential conflict of interest.

## Publisher's Note

All claims expressed in this article are solely those of the authors and do not necessarily represent those of their affiliated organizations, or those of the publisher, the editors and the reviewers. Any product that may be evaluated in this article, or claim that may be made by its manufacturer, is not guaranteed or endorsed by the publisher.

## Footnotes

^1^Propositions could for instance be modeled as sets of possible worlds, i.e., subsets of some fixed underlying set Ω of possible worlds. This “semantic” or “set-theoretic” notion of proposition is common in rational-choice theory and probability theory, where propositions are usually called “events.” Technically, *X* should be non-empty and closed under negation, i.e., a union of disjoint pairs of a proposition and its negation (The negation of a semantic proposition is of course its set-theoretic complement).

^2^The theorem assumes that the set *X* of propositions under consideration contains enough interconnections. Unsurprisingly, there is no impossibility of holding both belief types relative to *X* if *X* contains only one (contingent) proposition-negation pair *p*, ¬*p*, or more generally if all such pairs in *X* are logically independent, because holding consistent and deductively closed belief sets is trivial for such *X*. However the impossibility result does for instance apply under the standard assumption that the set of propositions *X* forms a Boolean algebra that is not trivially small, i.e., contains more than one (contingent) proposition-negation pair. Boolean algebras are usually taken for granted. Interestingly, they are not essential for the theorem. For details, we refer to Dietrich and List (2021).
